# Cell-free prototyping strategies for enhancing the sustainable production of polyhydroxyalkanoates bioplastics

**DOI:** 10.1093/synbio/ysy016

**Published:** 2018-09-04

**Authors:** Richard Kelwick, Luca Ricci, Soo Mei Chee, David Bell, Alexander J Webb, Paul S Freemont

**Affiliations:** 1 Section of Structural Biology, Department of Medicine, Imperial College London, London, UK; 2 Centre for Synthetic Biology and Innovation, Imperial College London, London, UK; 3 Department of Life Sciences and Systems Biology, University of Turin, Turin, Italy; 4 The London DNA Foundry, Imperial College London, London, UK

**Keywords:** synthetic biology, cell-free transcription–translation, polyhydroxyalkanoates, biopolymers, whey permeate

## Abstract

The polyhydroxyalkanoates (PHAs) are microbially-produced biopolymers that could potentially be used as sustainable alternatives to oil-derived plastics. However, PHAs are currently more expensive to produce than oil-derived plastics. Therefore, more efficient production processes would be desirable. Cell-free metabolic engineering strategies have already been used to optimize several biosynthetic pathways and we envisioned that cell-free strategies could be used for optimizing PHAs biosynthetic pathways. To this end, we developed several *Escherichia coli* cell-free systems for *in vitro* prototyping PHAs biosynthetic operons, and also for screening relevant metabolite recycling enzymes. Furthermore, we customized our cell-free reactions through the addition of whey permeate, an industrial waste that has been previously used to optimize *in vivo* PHAs production. We found that the inclusion of an optimal concentration of whey permeate enhanced relative cell-free GFPmut3b production by approximately 50%. In cell-free transcription–translation prototyping reactions, gas chromatography–mass spectrometry quantification of cell-free 3-hydroxybutyrate (3HB) production revealed differences between the activities of the Native **Δ**PhaC_C319A (1.18 ± 0.39 µM), C104 **Δ**PhaC_C319A (4.62 ± 1.31 µM) and C101 **Δ**PhaC_C319A (2.65 ± 1.27 µM) *phaCAB* operons that were tested. Interestingly, the most active operon, C104 produced higher levels of PHAs (or PHAs monomers) than the Native *phaCAB* operon in both *in vitro* and *in vivo* assays. Coupled cell-free biotransformation/transcription–translation reactions produced greater yields of 3HB (32.87 ± 6.58 µM), and these reactions were also used to characterize a *Clostridium propionicum* Acetyl-CoA recycling enzyme. Together, these data demonstrate that cell-free approaches complement *in vivo* workflows for identifying additional strategies for optimizing PHAs production.

## 1. Introduction

The mass production of oil-derived plastics has resulted in widespread and potentially irreversible global ecological damage (1, 2). Despite these environmental consequences, oil-derived plastics are still highly-demanded, due to their versatility and low-cost of production. Indeed, the demand for oil-derived plastics is sufficiently strong that even current recycling processes are not likely to influence the future levels of *de novo* plastic production (3). Moreover, the societal and technological challenges that are associated with the introduction of more sustainable alternatives to oil-derived plastics are significant, several of which have been extensively reviewed (4, 5). Synthetic biology strategies along with best practices in responsible research and innovation may help efforts to introduce sustainable alternatives to oil-derived plastics. Synthetic biology is a field that is driven by the development and carefully-considered implementation of rationally-engineered biotechnologies that might help to address local or global challenges (6–10). Developments in synthetic biology, along with a continuum of advancements in metabolic engineering, could potentially enable the routine and highly scalable production of microbially-produced biopolymers, such as polyhydroxyalkanoates (PHAs) (11–15). PHAs share several material characteristics with some of the most widely used oil-derived plastics, but beneficially, PHAs are also biodegradable (16, 17). In order to develop PHAs as viable alternatives to oil-derived plastics, great efforts have been undertaken to design more efficient microbial PHAs production processes through the rational engineering of biosynthetic pathways (13, 18–20), metabolite recycling processes (e.g. Acetyl-CoA) (21, 22) and the use of industrially-sourced, low-cost feedstocks (e.g. whey permeate) (23–28). Alternative, enzymatic approaches for the production of PHAs have also been investigated (29–31).

From these extensive studies, we reasoned that cell-free transcription–translation (TX-TL) systems could be used to efficiently characterize different PHAs biosynthetic operons. Indeed, cell-free TX-TL systems have been widely used as prototyping platforms for characterizing DNA based parts, devices and systems including: DNA regulatory elements (32–35), complex genetic circuits (36–40), cell-free medical biosensors (41–43) and biomaterials (44). These cell-free applications have also renewed interest in cell-free metabolic engineering approaches for *in vitro* enzyme screening and prototyping of entire biosynthetic pathways (45–51). Importantly, extract-based cell-free systems enable the *in situ* characterization of enzymes and biosynthetic pathways within a metabolic context that is representative of the production strain (52). Since cell extracts contain not only the TX-TL machinery needed to express a biosynthetic operon but also relevant enzyme co-factors and potentially competing metabolic pathways, cell-free metabolic engineering approaches can be used to identify novel strategies that improve the *in vivo* activities of biosynthetic pathways (53). Yet, interestingly cell-free TX-TL systems have not yet been used to characterize PHAs biosynthetic pathways. To this end, we developed several cell-free approaches for prototyping PHAs biosynthetic operons and relevant metabolite recycling enzymes (e.g. Acetyl-CoA). We also optimized a gas chromatography–mass spectrometry (GC-MS) method that can detect low concentrations of 3-hydroxybutyrate (3HB) (a PHAs monomer), in small-scale cell-free prototyping reactions. Furthermore, these cell-free reactions were enhanced through the addition of whey permeate, a low-cost industrial waste that has been previously used to optimize *in vivo* PHAs production. In combination these cell-free prototyping strategies complement *in vivo* workflows for identifying additional strategies for optimizing PHAs production.

## 2. Materials and methods

### 2.1 Bacterial strains

All constructs and bacterial strains used in this study are listed in Supplementary Table S1. *Escherichia coli* NEB10-beta was used for cloning, whilst *E. coli* MG1655 was used for experiments. For plasmid recovery *E. coli strains* were grown in Luria-Bertani (LB) supplemented with either 34 μg/ml Chloramphenicol (final concentration), 100 μg/ml Ampicillin (final concentration) or 50 μg/ml Kanamycin (final concentration), as appropriate, at 37°C, with shaking (220 rpm). 

### 2.2 Construct assembly

The empty vector negative control constructs EV101 (BBa_J23101; pRK21) and EV104 (BBa_K608002; pRK4) were originally sourced from the 2013 distribution of the iGEM Registry of Standard Biological Parts (partsregistry.org). EV104 was transformed into chemically competent *E. coli* MG1655 to create strain S_RK001. EV101 was transformed into chemically competent *E. coli* MG1655 to create strain S_RK008.

#### phaCAB bioplastic operon constructs

The Native-*phaCAB* construct (BBa_K934001; pRK51) was transformed into *E. coli* MG1655 to create strain, S_RK002. The constitutive C104 *phaCAB* operon construct (C104, BBa_K1149052; pRK61) was transformed into *E. coli* MG1655 to create strain, S_RK003. The Native (pRK51) and C104 (pRK61) *phaCAB* constructs are also available in the iGEM Registry of Standard Biological Parts (partsregistry.org) and were also reported in our previous study (13). The constitutive C101 *phaCAB* operon construct (C101—pRK14) is derived from C104, such that PCR (primer pairs RK024 CH101F and RK025 CH101R) was used to change the Anderson constitutive promoter to J23101. This new construct was transformed into *E. coli* MG1655 to create strain, S_RK006.

To generate constitutive *phaCAB*-operons with an inactive PhaC enzyme, PCR reactions were undertaken using PCR primer pairs PhaCCys319Ala_F (RK007) and PhaCCys319Ala_R (RK008) with Native (pRK51), C104 (pRK61) or C101 (pRK14) as the DNA template. The resultant PCR products were digested with DpnI restriction enzyme, phosphorylated, self-ligated (Quick Ligase, New England Biolabs, UK) and transformed into *E. coli* NEB10-beta, resulting in *phaCAB*-operon strain/plasmids, Native **Δ**PhaC_C319A (pRK13), C104 **Δ**PhaC_C319A (pRK7) and C101 **Δ**PhaC_C319A (pRK15). These plasmids were then purified from *E. coli* NEB10-beta using the Qiaprep Spin Miniprep Kit (Qiagen, Germany) and transformed into *E. coli* MG1655 competent cells, resulting in *E. coli* MG1655 strains S_RK004.

To generate the GFP expression plasmid (101_GFP), plasmid pRK1 was digested with XbaI and PstI to generate a fragment containing the RBS B0034, *gfpmut3b* and terminators B0010 and B0012. This fragment was ligated (Quick Ligase, New England Biolabs, UK) into a SpeI and PstI digested EV101 backbone (pRK3) and transformed into *E. coli* NEB10-beta to create plasmid/strain, pLR036.

#### pT7 constructs

A negative control, empty vector, encoding a T7 promoter (BBa_I719005; pRK9) was originally sourced from the 2013 distribution of the iGEM Registry of Standard Biological Parts (partsregistry.org).

Plasmid pT7_PCT (pRK12) was generated to enable inducible expression of the propionyl CoA transferase (PCT) gene from *Clostridium propionicum*. To generate the construct the *pct* gene sequence was obtained from GenBank (GenBank: AJ276553.1) and then synthesized as a GeneArt String DNA fragment (Life Technologies, USA). The GeneArt String DNA fragment was blunt-end cloned, using the Zero Blunt PCR Cloning Kit (Life Technologies, USA), into pCR-Blunt vector according to the manufacturer’s instructions—producing plasmid (pRK11). Afterwards, a PCR reaction was carried out using primers F1_PCT-FW (RK011) and F1_PCT-RV (RK012), where plasmid pRK11 served as the DNA template. The resultant PCR product generated a *pct* encoding DNA fragment. Additionally, a PCR reaction was carried out using primers T7_INF_FWD (RK009) and T7_INF_REV (RK010), where plasmid pRK10 served as the DNA template. The resultant PCR product generated a linearized plasmid vector. The PCR products from both reactions were used in an In-Fusion cloning reaction (Takara Bio, USA) and 2.5 µl of the completed reaction was transformed into NEB10-beta, producing strain/plasmid pRK12.

The DNA sequences of all inserts/constructs were verified using the sequencing service provided by Eurofins Genomics GmbH (Ebersberg, Germany), which generally provided sequencing reads of >800 bp. Sequencing chromatograms were checked using SnapGene software (v4.1), to ensure quality, and sequencing results were aligned using Serial Cloner (v2-6-1) software alignment tool against reference sequences in order to confirm that there were no discrepancies between cloned and expected sequences. Inserts were fully sequenced. Primers used for sequencing and cloning are listed in Supplementary Table S2 and are provided as Supplementary FASTA files. Plasmid maps and GenBank files that also display the locations of sequencing primers are included as Supplementary files.

### 2.3 Preparation of cell extracts

To prepare cell-free extracts, *E. coli* MG1655 cells were revived from glycerol stocks onto 2× YTP plates (31 g/l 2× YT, 40 mM potassium phosphate dibasic, 22 mM potassium phosphate monobasic and 15 g/l agar). Once streaked, plates were incubated overnight at 37°C. Individual colonies were inoculated into 5 ml 2× YTP medium (31 g/l 2× YT, 40 mM potassium phosphate dibasic and 22 mM potassium phosphate monobasic) and incubated overnight with shaking (220 rpm) at 37°C. 2.5 ml aliquots of the resultant cultures were used to inoculate flasks containing 50 ml 2× YTP medium. These cultures were then incubated at 37°C with shaking (220 rpm) until the cell density reached an OD600_nm_ of two. Finally, 25 ml of the resultant cultures were used to inoculate flasks containing 500 ml 2× YTP medium. These cultures were subsequently incubated at 37°C with shaking (220 rpm) until cell density reached an OD600_nm_ of between 2 and 3. For the preparation of cell extracts from strain S_RK004, the cells were cultured using these same conditions except that 2× YTP plates and media were also supplemented with 34 µg/ml Chloramphenicol.

To harvest cells, 500 ml cultures were centrifuged at 3220 *g* for 15 min. Cell pellets were re-suspended into 20 ml S30-A buffer [14 mM Magnesium (Mg) glutamate, 60 mM Potassium (K) glutamate, 50 mM Tris, 2 mM 1,4-Dithiothreitol (DTT), pH 7.7] and transferred into a pre-weighed 50 ml Falcon tube. Each 50 ml Falcon tube was centrifuged (2000 *g*, 10 min, 4°C), pellets washed with 20 ml S30-A buffer and subsequently re-centrifuged (2000 *g*, 10 min, 4°C) to form the final cell pellets in preparation for cell lysis. To determine the weight of the cell pellet, the weight of the 50 ml falcon tube was subtracted from the combined weight of the 50 ml tube and cell pellet. Pellets were stored at −80°C for no more than 48 h, prior to cell lysis.

To lyse the cells, pellets were defrosted on ice and re-suspended into 1 ml S30-A buffer per gram of cell pellet and aliquoted as 1 ml samples in 1.5 ml microtubes. Samples were sonicated on ice (3 × 40 s with 1-min cooling interval; output frequency: 20 kHz; amplitude: 50%) and then centrifuged (12 000 *g* at 4°C for 10 min). The supernatants were removed, aliquoted at 500 μl into 2 ml screw cap tubes and incubated with shaking (220 rpm) at 37°C for 80 min. Post pre-incubation, samples were stored on ice and then centrifuged (12 000 *g* at 4°C for 10 min). Supernatants were removed and aliquoted into dialysis cassettes (GeBAflex-Maxi Dialysis Tubes—8 kDa MWCO, Generon) for dialysis into S30-B buffer (14 mM Mg-glutamate, 60 mM K-glutamate, 5 mM Tris, 1 mM DTT; pH 8.2) with stirring at 4°C for 3 h. Post-dialysis samples were centrifuged (12 000 *g* at 4°C for 10 min), the extract supernatants were aliquoted into 1.5 ml tubes, flash frozen in liquid nitrogen and stored at −80°C for use in cell-free reactions.

### 2.4 Cell-free transcription–translation reactions

Cell-free transcription–translation reactions consisted of three parts mixed together in the indicated ratios: cell extract (33% v/v), energy buffer (42% v/v) and plasmid DNA (25% v/v). The final reaction conditions were: 8 mM Mg-glutamate, 260 mM K-glutamate, 1.5 mM each amino acid (except leucine—1.25 mM leucine), 1.5 mM of both ATP and GTP, 0.9 mM of both CTP and UTP, 1.5 mM spermidine, 0–5.748 g/l of Molkolac instant demineralized whey permeate (Orchard Valley Food Ingredients, UK) or 0–4.836 g/l lactose (Sigma Aldrich, MO, USA, #L3625-1KG), 0 or 100 μM (final concentration) SNARF-5F pH sensitive dye (Invitrogen, USA) and 10 nM (final concentration) plasmid DNA. For analysis of cell-free GFP production or pH, 10 μl cell-free reactions were aliquoted into individual wells of 384-well plates (Griener bio-one, NC, USA) and measured using a Clariostar plate reader (BMG, UK) with the following settings: GFP—excitation 483–14 nm and emission 530–30 nm, for pH—excitation 514 nm and the ratio of two different emissions, 580/640 nm were measured and used in conjunction with a SNARF-5F calibration curve (Supplementary Figure S1). Plates were sealed, shaken prior to each reading cycle (5 seconds, 500 rpm, orbital) and the plate reader was set to incubate the cell-free reactions at 30°C. Scaled up 30 μl cell-free GFP production reactions were incubated in 1.5 ml tubes at 30°C for 5 h. 10 μl of these cell-free reactions were then aliquoted into individual wells of a 384-well plate and measured using a Clariostar plate reader using the GFP settings described above.

Coupled cell-free biotransformation with transcription–translation reactions were setup similarly to cell-free transcription–translation reactions, except that they were based on cell extracts from strain S_RK004, which contains phaCAB enzymes in the cell extract (where PhaC is catalytically inactive). Also, these reactions included 0 or 0.004 g/l Molkolac instant demineralized whey permeate, 0 (−T7) or 25 (+T7) units of T7 RNA polymerase and 0 or 10 nM (final concentration) of pT7_PCT (pRK12) plasmid DNA. These reactions were setup as 30 μl reactions in 1.5 ml tubes at 30°C for 0–5 h.

For GC-MS analysis of 3HB content in cell-free reactions, the reactions were scaled up to 30 μl and were incubated in 1.5 ml tubes at 30°C for 5 h. Cell-free reactions were then treated with 30 μl ice cold acetonitrile, centrifuged (12 000 *g* at 4°C for 10 min) and the resultant supernatants were used for downstream GC-MS analysis. For analysis of Acetyl-CoA content in cell-free reactions the reactions were scaled up to 30 μl and were incubated in 1.5 ml tubes at 30°C for 0–2.5 h. At the indicated timepoint these samples were then deproteinized according to the manufacturer’s instructions in the PicoProbe Acetyl-CoA Fluorometric Assay Kit (Abcam, UK). Briefly, cell-free samples were deproteinized using perchloric acid then neutralized with 3 M KHCO_3_ as per the kits instructions.

### 2.5 SNARF-5F pH calibration curve

A pH-sensitive calibration curve was generated using the SNARF-5F dye, 5-(and-6)-carboxylic acid (Invitrogen, USA). The dye was dissolved in DMSO at 1 mM and stored at +4°C. SNARF-5F was diluted to 100 μM (final concentration) in 10 μl (total volume) of a range of different 100 mM Tris buffers (final concentration) that were set, using 0.5 M acetic acid, at a range of defined pH strengths. 10 μl aliquots of these mixtures were aliquoted into individual wells of a 384-well plate (Greiner bio-one, NC, USA). End-point fluorescence measurements were carried out using a Clariostar plate reader (BMG, UK) set to an excitation of 514 nm and a ratio of two different emissions, 580/640 nm. From the calibration curve a third order polynomial fitting was developed to extrapolate the pH values from the fluorescence 580/640 nm emissions ratio. These calibration curve data are shown in Supplementary Figure S1.

### 2.6 3-Hydroxybutyric acid detection in cell-free samples using GC-MS

The cell free samples were subjected to a trimethylsilylation reaction for the detection of 3HB production using GC-MS. The 3HB samples were centrifuged for 5 min at 13 500 rpm, then 40 µl of the supernatant was dried under a gentle nitrogen stream.

To the dried samples was added 90 µl of MSTFA and MSTFA + 1% TMCS silylation reagent (Thermo Fisher, part number: 11567851) and left to react at 37°C for 30 min. The commercial 3HB standard (Sigma Aldrich, #54965-10G-F) was dissolved in methanol (Sigma Aldrich) at several different concentrations between the range of 0 µM and 200 µM in serial dilutions to generate a calibration curve (Supplementary Figure S4). 10 µl of the standards were then evaporated to dryness under a gentle nitrogen stream. The dried standards were treated with the trimethylsilylation reaction as described above.

The trimethylsilylated derivatized samples were analysed by GC-MS, using an Agilent Technologies 7890B GC and MSD 5977 series system with electron ionization in SIM mode by monitoring ion with m/z value 117.1, 147.1, 191.1 and 233.0. Helium was used as the carrier gas. The temperatures of the injector and MS transfer line were 240°C and 250°C, respectively, whereas MS quadrupole and MS source were 150°C and 250°C, respectively. The samples were analysed with an injection volume of 1 µl, at a split ratio of 10 to 1. A temperature programme was used for separation of the trimethylsilylated 3HB: initial temperature is 80°C, temperature increase 30°C/min until 123°C, temperature increase 1°C/min until 128°C, temperature increase 60°C/min until 280°C, and hold for 3 min, followed by 0.5 min post run at 80°C.

### 2.7 Acetyl-CoA assay

Acetyl-CoA content was quantified using the PicoProbe acetyl-CoA assay kit (ab87546, Abcam, UK). The 0–100 pM range acetyl-CoA standard curve was generated with a correlation coefficient of 0.9982 (Supplementary Figure S3). In order to correct for background (free CoASH and succ-CoA) in cell-free samples, as per the manufacturer’s instructions, CoASH Quencher and Quencher remover were used. Samples were then diluted with the reaction mix and fluorescence was measured (Excitation 535, Emission 589 nm) using a Clariostar plate reader (BMG, UK).

### 2.8 Nile red plate assay

Nile red plate assays for qualitative detection of PHAs were carried out as previously described (13). Briefly, *E. coli* MG1655 transformed with either a negative control plasmid (EV104 or EV101) or a *phaCAB* operon (Native, C104, C101 or **Δ**PhaC_C319A inactive PhaC operon versions) were grown in 5 ml 2×YT+P media (supplemented with 34 μg/ml Chloramphenicol) overnight at 37°C with shaking (220 rpm). Liquid cultures were then streaked onto 2×YT+P-agar plates supplemented with 34 μg/ml Chloramphenicol, 0.5 μg/ml of Nile Red stain (Sigma-Aldrich, #72485–100MG) in 100% DMSO (v/v) and 120.48 g/l whey permeate. Nile red plates were incubated for 24 h at 37°C and imaged using a Fuji Film LAS-5000 imager (Ex. 473 and Em. Cy5 filter).

### 2.9 Flow cytometry analysis of PHAs production

Flow cytometry analysis of PHAs content was carried out similarly to previous reports (13, 54). Briefly, *E. coli* MG1655 harbouring either a negative control plasmid (EV104) or a *phaCAB* operon (Native, C104, or C104 **Δ**PhaC_C319A) were grown overnight at 37°C in 5 ml of 2×YT+P medium supplemented with 34 μg/ml Chloramphenicol. The resultant cultures were diluted to an OD_600nm_ of 0.8 in 6 ml of PHAs production media; 2×YT+P supplemented with 34 μg/ml Chloramphenicol and 120.48 g/l Molkolac instant demineralized whey permeate (Orchard Valley Food Ingredients, UK) or 3% glucose (w/v), and were cultured at 37°C for 24 h. Subsequently, 1 ml of each culture was centrifuged (7200 *g*), washed with 1 ml 1× PBS, and fixed with 35% ethanol [v/v] at room temperature for 15 min. Post-fixation, cultures were centrifuged (7200 *g*), re-suspended in 1 ml 1× PBS and stained with Nile Red (Sigma-Aldrich, #72485–100MG) to a final concentration of 20 μg/ml (in 100% DMSO) for 10 min on ice. Nile red stained *E. coli* were diluted (1:100) into 1× PBS before being loaded into an Attune NxT (ThermoFisher Scientific, MA, USA) flow cytometer. PHAs content was determined via flow cytometry analysis of Nile Red staining (YL2-A+, Ex. 560 nm, Em. 610 nm). Flow cytometry data analysis was carried out using FlowJo (v 10.1) software. Doublets were removed during gating. The background signal, as determined by the average geometric mean (YL2-A) of the appropriate, Nile red stained, empty vector transformed *E. coli* was removed and these data were normalized to Native-*phaCAB* engineered *E. coli.*

### 2.10 PHAs purification

PHAs purification was carried out using a scaled down version of a previously reported sodium hypochlorite-based method (13, 55). Briefly, glycerol stocks of *E. coli* MG1655 strains harboring either a negative control plasmid (EV104) or a *phaCAB*-operon (Native, or C104) were used to inoculate flasks containing 30 ml of 2×YT+P medium (supplemented with 34 μg/ml Chloramphenicol). These starter cultures were grown overnight, with shaking (220 rpm), at 37°C. The resultant cultures were then diluted to an OD_600nm_ of 0.8 in 100 ml of PHAs production media (2×YT+P media supplemented with 120.48 g/l whey permeate and 34 μg/ml Chloramphenicol (final concentration)). These PHAs production cultures were then incubated at 37°C, with 220 rpm shaking, for 24 h. Subsequently, 100 ml PHAs production cultures were centrifuged at 4200 rpm (Beckman J6-M1, USA) for 20 min. Post-centrifugation, bacterial cell pellets were re-suspended in 1× PBS, transferred into pre-weighed 50 ml tubes and centrifuged at 3220 *g* for 15 min at 4°C. The supernatant from each 50 ml tube was removed and the cell pellets were dried at 70°C for 60 min and weighed. Dried cell pellets were re-suspended in 10 ml 1× PBS, centrifuged at 3220 *g* for 15 min at 4°C, and the supernatant was discarded. Cell pellets were then suspended in 1× PBS with 1% Triton-X 100 (v/v in PBS) and then incubated for 30 min at room temperature. For the final PHA purification steps, cells were centrifuged at 3220 *g* for 15 min at 4°C, washed with 1× PBS and then incubated with 12 ml aqueous sodium hypochlorite for 80 min at 30°C with 220 rpm shaking. The resultant purified PHAs granules were centrifuged at 3220 *g* for 30 min at room temperature, washed with distilled water and dried overnight at 37°C and then 2 h at 70°C. To determine the weight of the dry cell pellets, the weight of the 50 ml tube was subtracted from the combined weight of the 50 ml tube and dry cell pellet. To determine the weight of the purified PHAs, the weight of the 50 ml tube was subtracted from the combined weight of the 50 ml tube and PHAs.

### 2.11 Monomer identification of *in vivo* produced PHAs using GC-MS

The poly-3-hydroxybutyrate (P(3HB)) samples were subjected to methanolysis in a solution of 425 µl of 100% methanol, 75 µl of sulphuric acid (6.6M) and 500 µl of dichloromethane in a small screw-top test tube and left to react for 3 h at 100°C. After allowing the mixture to cool, 500 µl of dichloromethane and 1 ml of water were added; the mixture shaken vigorously for 1 min followed by centrifugation at 4000 rpm for 5 min. The organic phase was removed and transferred to a screw-cap glass vial. GC-MS analysis was performed on an Agilent Technologies 7890B GC and MSD 5977 series system with electron ionization in scan mode. Helium was used as the carrier gas. The temperatures of the injector and MS transfer line were 240°C and 250°C, respectively, whereas MS quadrupole and MS source were 150°C and 230°C, respectively. The samples were analysed with an injection volume of 1 µl, at a split ratio of 14 to 1. A temperature program was used for efficient separation of the esters: 55°C for 3 min; temperature increase 15°C/min until 200°C and hold for 3 min.

## 3. Results and discussion

### 3.1 Development of a whey permeate-based cell-free energy system

Conventional cell-free TX-TL reactions are broadly composed of three main components: a cell extract, an energy mix and the DNA construct (e.g. plasmid or linear PCR product) that encodes the protein, RNA/DNA based device or biosynthetic pathway that is being tested. Simplified, and/or automated cell extract preparation methods have led to improvements in cell-free performance (56–58). Likewise, changes in the composition and/or concentrations of cell-free energy mix components have also improved cell-free performance, largely through improved ATP regeneration, the recycling of inorganic phosphate and through the use of inexpensive single (e.g. glucose) or dual energy sources (e.g. maltose or maltodextrin and glutamate) that make optimal use of central metabolic pathways (e.g. glycolysis and tricarboxylic acid cycle) (59–61). Cell-free energy mixes can also be simplified through the rational testing and removal of unnecessary components that upon re-examination, are largely historical artefacts from protocols that have now been superseded by improved cell-free methodologies. Such approaches have been used to develop minimal energy mixes that maintain cell-free performance but with seven fewer energy components than widely reported cell-free protocols (62).

Upon consideration of several cell-free methodologies, a fairly simplified cell extract preparation workflow was adapted from our previous cell-free work (34) and coupled with a recently reported glutamate-based minimal energy mix (62). Using these cell-free methods, several batches of *E. coli* MG1655 TX-TL cell extracts and minimal energy mix were generated (see Sections 2.3 and 2.4). These cell-free reactions were customized for the purposes of characterizing PHAs biosynthetic pathways through the inclusion of whey permeate. Whey permeate is a waste by-product of industrial cheese production that has been proposed as an attractive low-cost feedstock for microbial PHAs production *in vivo*—particularly P(3HB) (63, 64). Whey permeate contains a high percentage of lactose (>70% of the mass), which can be readily metabolized *in vivo* by *E. coli* MG1655, using its β-galactosidase enzyme, to generate glucose for glycolytic processing into Acetyl-CoA (65). Acetyl-CoA can then directly feed into *phaCAB* biosynthetic operons where it is enzymatically processed by PhaA (3-ketothiolase) to form acetoacetyl-CoA. Subsequently, PhaB (acetoacetyl-CoA reductase) reduces acetoacetyl-CoA to form (*R*)-3-hydroxybutyl-CoA ((*R*)-3HB-CoA), which is finally polymerized by PhaC (PHA synthase) to form the final PHAs polymer-P(3HB).

**Figure 1.**
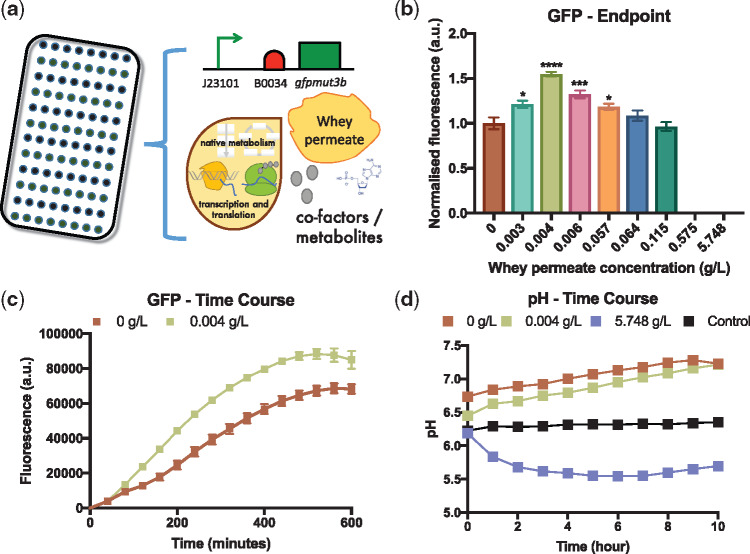
Whey permeate enhances cell-free protein synthesis. (**a**) Schematic of whey permeate enhanced cell-free transcription–translation reactions (**b**) Endpoint (5 h) analysis of cell-free GFP production in cell-free reactions with 0–5.748 g/l whey permeate. These data were normalized to the relative fluorescence of cell-free reactions containing 0 g/l whey permeate. (**c**) Time-course analysis of GFP production in cell-free reactions with 0 or 0.004 g/l of whey permeate. (**d**) Time-course analysis of pH in cell-free reactions which included 0–5.748 g/l of whey permeate. Control reactions were whey permeate without cell extract. Error bars denote standard error of the mean. Student’s *t*-test, *P < 0.05, ***P < 0.0001 and ****P < 0.0001, *n* = 3–9.

To determine whether whey permeate could be similarly metabolized *in vitro* a series of *E. coli* MG1655 cell-free TX-TL reactions were setup that included a minimal energy mix but with different concentrations of whey permeate (0–5.748 g/l) and a constitutive *gfpmut3b* expression plasmid (101_GFP) (Figure 1a). An endpoint (5 h) analysis of cell-free GFPmut3b production revealed that whey concentrations above 0.115 g/l completely inhibit cell-free protein synthesis (Figure 1b). It is likely that relatively high concentrations of whey permeate salts are responsible for this inhibitory effect (Supplementary Table S3). Indeed, previous reports have shown that the concentrations of different salts can significantly impact cell-free performance (66). We, therefore, used demineralized whey permeate, which has been processed to lower its salt and mineral content. Lower concentrations of whey permeate were not inhibitory and interestingly 0.004 g/l of whey permeate enhanced relative cell-free GFPmut3b production by approximately 50 ± 2.5% (Figure 1b). Cell-free dynamics, in terms of the rate of cell-free GFPmut3b production, were also enhanced in cell-free reactions with 0.004 g/l of whey permeate in comparison to reactions that included the standard minimal energy mix (0 g/l) (Figure 1c). An optimal concentration of whey permeate (0.004 g/l) also enhanced the cell-free production of GFPmut3b in slightly larger scale (30 µl) cell-free reactions (Supplementary Figure S2).

We hypothesized that the glycolytic processing of the lactose component of whey permeate is the most likely mechanism through which whey permeate enhances cell-free protein synthesis. Indeed, previous reports have shown that glucose and maltose are metabolized, through glycolysis, in cell-free extracts and that this glycolytic activity leads to the generation of ATP, which subsequently, enhances cell-free protein synthesis (59, 60). Previous studies have also shown that the pH of cell-free reactions can be a useful indicator of glycolytic activity in cell extracts. Essentially, increased rates of glycolysis in cell-free extracts lead to higher levels of lactate and acetate, which lower the pH (60). The pH of cell-free reactions can be measured using a pH sensitive fluorescent dye—such as SNARF-5F (see Section 2.5). Similar to a previous report, we setup a SNARF-5F calibration curve to convert between dye fluorescence and pH (Supplementary Figure S1) (60). The pH of cell-free reactions without whey permeate (0 g/l) was generally stable throughout the 10 h reaction (Figure 1d). Whereas the pH was slightly lower in cell-free TX-TL reactions with 0.004 g/l of whey permeate and the highest concentration tested (5.748 g/l) resulted in a significant pH drop from 6.2 to 5.6 (Figure 1d). Likewise, the addition of lactose (at an equivalent concentration to that found in whey permeate) also enhanced cell-free GFPmut3b production and the pH in these cell-free reactions also decreased over time (Supplementary Figure S3). As additional controls we also measured the pH of either whey permeate (5.748 g/l) or lactose (4.836 g/l) in reactions that included the minimal cell-free energy mix but that didn’t include cell extracts. Since these reactions didn’t include cell extracts, lactose was not metabolized. As expected, the pH of these reactions did not change (Figure 1d and Supplementary Figure S3), which is consistent with our hypothesis that whey permeate (lactose) likely enhances protein synthesis through glycolysis. Since an optimal concentration of whey permeate (0.004 g/l) enhanced cell-free protein synthesis it was included in subsequent prototyping experiments.

### 3.2 Cell-free transcription–translation reactions for prototyping PHAs biosynthetic operons

In our previous study, the Native *phaCAB* operon from *Cupriavidus necator* was used to engineer several *phaCAB* operon variants that enhanced *in vivo* PHAs production in *E. coli* (13). Amongst these variants, the C104 constitutive *phaCAB* operon (BBa_K1149052) was designed such that the native promoter and RBS were replaced by an Anderson constitutive promoter (BBa_J23104) and a strong synthetic ribosome binding site (RBS, BBa_B0034). Previously, the C104 design enhanced *in vivo* P(3HB) production from glucose by up to three-fold, in comparison to Native *phaCAB*-engineered *E. coli* (13). We also derived the C101 constitutive *phaCAB* operon (pRK14), from C104, in which PCR was used to change the Anderson constitutive promoter to J23101 (BBa_J23101). Since, these *phaCAB* operons produce different levels of P(3HB) *in vivo*, we reasoned that they would be useful constructs to test whether cell-free transcription–translation prototyping reactions could be used to identify and characterize differences between different *phaCAB* operons.

Initial attempts to detect P(3HB) production in cell-free prototyping reactions were based on several methods adapted from previously described liquid chromatography–mass spectrometry (LC-MS) and GC-MS approaches (67). However, these methods were found to be unreliable for detecting P(3HB) in cell-free TX-TL reactions. There are several reasons which could explain a lack of detection. Firstly, established PHAs analyses methods were largely designed for detecting highly concentrated and purified, high molecular weight PHAs polymers (68). Whereas, small-scale cell-free TX-TL prototyping reactions likely generate relatively low concentrations of PHAs at a range of molecular weights (polymer chain lengths). Secondly, the samples also contain a complex background of many different cell extract-derived metabolites that further complicate PHAs detection. Furthermore, additional processing steps that are required to depolymerize P(3HB) into the more easily detectable monomer form (3HB), may increase the risk of sample loss. Due to these challenges, additional methods development was carried out.

*Escherichia coli* MG1655 cell extracts were spiked with known concentrations of commercially available 3HB and these spiked extracts were analysed using different GC-MS methodologies. From these experiments, an optimized GC-MS method was developed, along with a calibration curve, which was used to detect 3HB in the low micromolar range within TX-TL cell extracts (Section 2.6; Supplementary Figure S4). Method optimization was achieved through improvements in the temperature gradient for better separation of derivatized 3HB from the cell extract background. A more sensitive and selective mass spectrometry (MS) acquisition was also acquired, using the selected ion monitoring (SIM) approach, coupled with monitoring *m*/*z* values, as described in the Section 2.6, during the GC-MS analysis. In parallel, we also engineered an additional variant for each of these *phaCAB* operons in which we used PCR to introduce a site-specific mutation within the *phaC* gene to ensure that the PhaC enzymes were catalytically inactive (**Δ**PhaC_C319A, cysteine 319 to alanine) and therefore unable to polymerize (*R*)-3HB-CoA into P(3HB). These operons were termed Native **Δ**PhaC_C319A (pRK13), C104 **Δ**PhaC_C319A (pRK7) and C101 **Δ**PhaC_C319A (pRK15), respectively. These additional operons were created to simplify sample processing for GC-MS and to allow the direct detection of 3HB (a PHAs monomer) in cell-free prototyping reactions.

**Figure 2.**
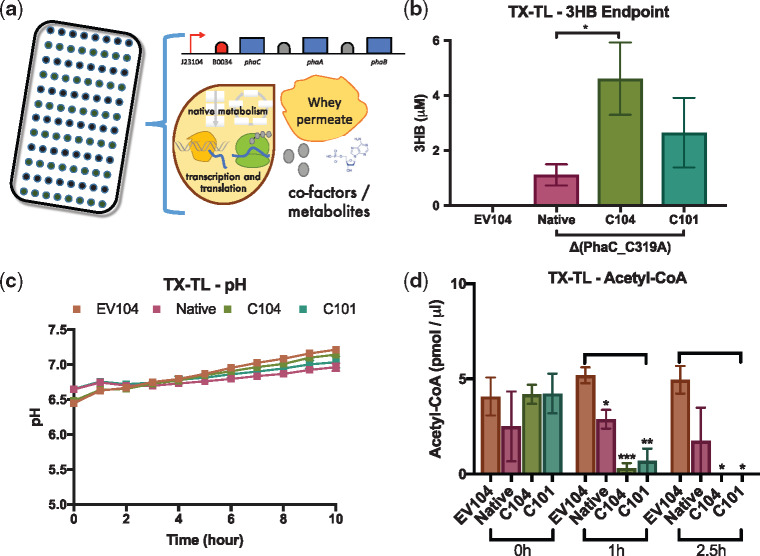
Cell-free characterization of *phaCAB* biosynthetic operons. (**a**) Schematic of whey permeate enhanced cell-free transcription–translation reactions for prototyping *phaCAB* operons. (**b**) Endpoint (5 h) GC-MS analysis of 3HB production in cell-free *phaCAB* operon prototyping reactions. (**c**) Time-course analysis of pH in cell-free *phaCAB* prototyping reactions. (**d**) Endpoint analysis of Acetyl-CoA content in cell-free *phaCAB* prototyping reactions. Error bars denote standard error of the mean. Student’s *t*-test, *P < 0.05, **P < 0.01 and ***P < 0.0001, *n* = 3–9.

Cell-free prototyping reactions were setup using three different cell extract batches and analysed using our optimized GC-MS method. In cell-free transcription–translation prototyping reactions, GC-MS quantification of cell-free 3HB production revealed differences between the activities of the Native **Δ**PhaC_C319A (1.18 ± 0.39 µM), C104 **Δ**PhaC_C319A (4.62 ± 1.31 µM) and C101 **Δ**PhaC_C319A (2.65 ± 1.27 µM) *phaCAB* operons that were tested (Figure 2b). In negative control reactions (EV104) 3HB was not detectable (Figure 2b).

A pH analysis of negative control (EV104) and *phaCAB* prototyping reactions (Native, C104 and C101) confirmed that, as expected, the pH of these cell-free transcription–translation reactions is largely determined by whey permeate metabolism and not by the activities of the PhaCAB enzymes (Figure 2c). In order to gain more direct insights into the activities of *phaCAB* operons in cell-free prototyping reactions we assayed endpoint Acetyl-CoA content at 0, 1 and 2.5 h, respectively (Figure 2d). We reasoned that the rate in which Acetyl-CoA is converted towards P(3HB) in cell-free prototyping reactions might differ between different *phaCAB* operons. To test this, we setup whey permeate enhanced cell-free prototyping reactions that either included a negative control plasmid (EV104) or a *phaCAB* operon (Native, C104 or C101). Interestingly, at both 1 h and 2.5 h, cell-free *phaCAB* prototyping reactions had lower Acetyl-CoA content than the negative control reactions (Figure 2d).

Together, these data demonstrate that 3HB (PHAs monomer) can be produced and detected within cell-free prototyping reactions. Whilst it is easier to detect PHAs monomers in these cell-free prototyping reactions, we were still able to utilize *phaCAB* operons that included active PhaC enzymes in our other assays (pH and acetyl-CoA). Whilst we observed some variation between Acetyl-CoA assay replicates, these data indicate that these types of cell-free analyses can be used to screen for differences in the activities of different *phaCAB* operons. Indeed, the general pattern in these Acetyl-CoA assay data indicate that the C104 and C101 *phaCAB* operons are likely to enhance *in vivo* PHAs production, relative to the Native *phaCAB* operon.

### 3.3 Biotransformation of whey permeate in *phaCAB*-engineered cell extracts

Extract-based cell-free metabolic engineering (also known as cell-free protein synthesis driven metabolic engineering, CFPS-ME) can be categorized into two main strategies; one that makes use of cell-free transcription and translation systems and the other involving mixing cell extracts (49). Such strategies use cell-free TX-TL systems for *in vitro* enzyme production and expression of entire biosynthetic pathways. Cell-free metabolic engineering can also be used to construct biosynthetic pathways through the rational mixing of different cell extracts (lysates) that include the necessary enzymes, co-factors and metabolic pathways. It is possible to combine these different cell-free strategies. For instance, bacterial strains that produce desirable enzymes during cell growth can later be lysed and processed to generate cell extracts for use in cell-free transcription–translation reactions. Therefore, a combined cell-free metabolic engineering approach could be used as a prototyping platform for the *in vitro* characterization of additional enzymes that may directly or indirectly enhance PHAs production (e.g. metabolite recycling enzymes).

**Figure 3.**
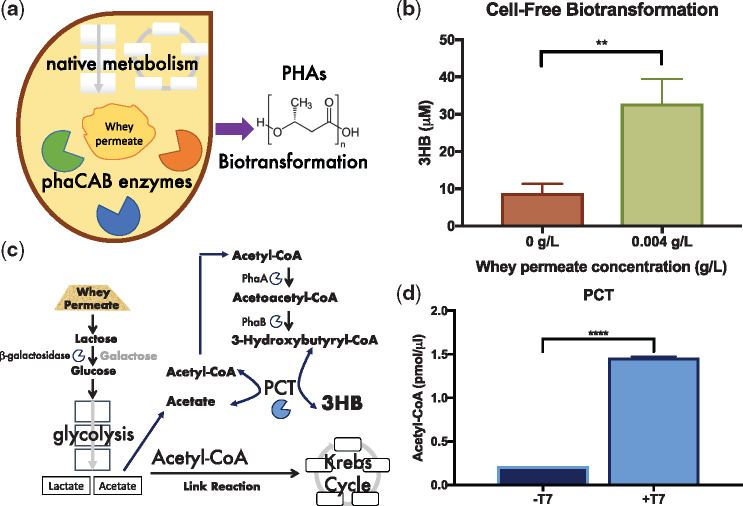
Coupled cell-free biotransformation and transcription–translation reactions (**a**) Schematic of whey permeate enhanced cell-free biotransformation reactions. (**b**) Endpoint (5 h) GC-MS analysis of 3HB production in cell-free biotransformation reactions. (**c**) Schematic depicts the Acetyl-CoA recycling activity of propionyl CoA transferase (PCT) within the context of whey permeate metabolism in cell extracts. (**d**) End point (2.5 h) analysis of Acetyl-CoA content in coupled cell-free biotransformation and transcription–translation reactions. These reactions also included a T7 inducible *pct* expression plasmid with (+T7) or without (−T7) T7 RNA polymerase. Error bars denote standard error of the mean. Student’s *t*-test, **P < 0.01 and ****P < 0.0001, *n* = 3–6.

In order to test the concept of cell-free biotransformation reactions, several batches of cell extract from C104 **Δ**PhaC_C319A *phaCAB*-engineered *E. coli* MG1655 (S_RK004) were generated. These cell extracts include endogenous β-galactosidase, central metabolic pathways (e.g. glycolysis) and all three PhaCAB enzymes (inactive PhaC), and thus contain all the enzymes needed for the biotransformation of whey permeate into 3HB (PHAs monomers) (Figure 3a). Biotransformation reactions were carried out as described in Section 2. Endpoint (5 h) GC-MS analyses of the cell-free biotransformation reactions revealed that average 3HB content was 8.84 ± 2.47 µM in reactions with 0 g/l of whey permeate and 32.87 ± 6.58 µM in reactions with 0.004 g/l of whey permeate (Figure 3b). Even those cell-free biotransformation reactions without an exogenously added glycolysis substrate (0 g/l whey permeate), produced 3HB (Figure 3b), suggesting that these cell extracts might be able to generate Acetyl-CoA using an alternative pathway, such as through β-oxidation (69). Interestingly, cell-free production of 3HB from whey permeate (0.004 g/l) was around 7× higher in biotransformation reactions (Figure 3b) than in TX-TL reactions (Figure 2b). We anticipate that, with further developments in strain engineering, cell-free biotransformation reactions could conceivably be used for PHAs production. Of course, cell-free biotransformation reactions are not limited to PHAs production applications and can potentially be combined with cell-free TX-TL approaches for screening enzymes that might enhance PHAs production in both cell-free and *in vivo* contexts.

As an exemplar, we tested the cell-free expression of the propionyl CoA transferase (*pct*) gene from *C. propionicum* in C104 **Δ**PhaC_C319A *phaCAB*-engineered cell extracts. PCT couples the release of CoA from (*R*)-3HB-CoA with the generation of Acetyl-CoA from acetate (Figure 3c) (22, 70). Since, whey permeate is likely to increase the glycolytic production of acetate and PCT reactions generate Acetyl-CoA (a precursor of 3HB), it was anticipated that Acetyl-CoA and 3HB levels would be relatively higher in cell-free biotransformation reactions that express the *pct* gene. The *C. propionicum pct* gene sequence was sourced from Genbank (AJ276553.1) and cloned into a T7 expression plasmid (as described in Section 2.2), which was termed T7_PCT (pRK12). Combined biotransformation/TX-TL cell-free reactions were setup with C104 **Δ**PhaC_C319A *phaCAB*-engineered cell extracts, in combination with the T7_PCT plasmid, and either 0 (−T7) or 25 (+T7) units of T7 RNA polymerase (Section 2.5). Thus, only those cell-free reactions that contain T7 RNA polymerase will express the *pct* gene. Endpoint analyses revealed that average Acetyl-CoA content was higher in cell-free reactions that contained T7 RNA polymerase (+T7—1.46 ± 0.01 pmol/µl) in comparison to control reactions with no T7 RNA polymerase (−T7—0.21 ± 0.00 pmol/µl) (Figure 3d). Whilst the PCT driven increase in Acetyl-CoA is relatively modest and did not result in increased 3HB content (Supplementary Table S4), these data suggest that coupled cell-free biotransformation with TX-TL reactions could be useful in helping to identify metabolite recycling enzymes that might improve PHAs production *in vivo*. Indeed, in other experimental contexts, *pct* genes from both *C. propionicum* and *Megasphaera elsdenii* have been shown to enhance *in vivo* Acetyl-CoA content (22, 70–72).

### 3.4 *In vivo* characterization of PHAs production from whey permeate

Finally, *in vivo* PHAs production experiments were carried out. The optimal whey permeate concentration for cell-free assays (0.004 g/l, Figure 1b) is likely to be insufficient for *in vivo* PHAs production, where a relative excess of carbon is desirable. Therefore, a previously described whey permeate concentration of 120.48 g/l, corresponding to 100 g/l of lactose, was used in all *in vivo* assays (63).

**Figure 4.**
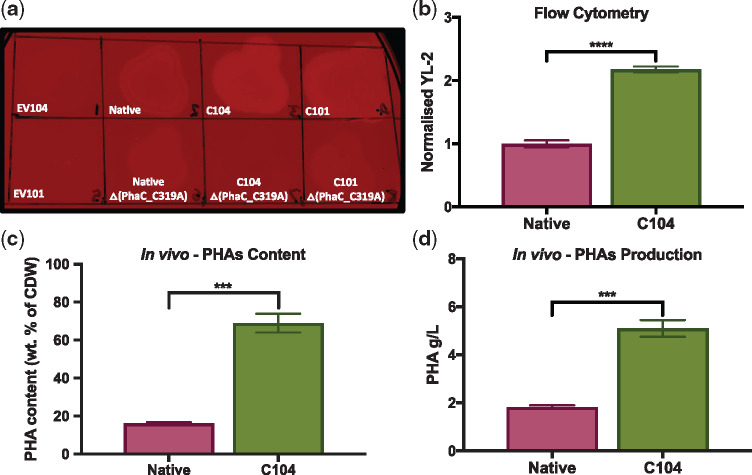
*In vivo* characterization of PHAs production from whey permeate in *phaCAB*-engineered *E. coli.* (**a**) Nile red plate assay for qualitative detection of *in vivo* PHAs production in control and *phaCAB* engineered *E. coli* (**b**) Flow cytometry analysis of *in vivo* PHAs production in Nile red stained, *phaCAB* engineered *E. coli.* The background signal, as determined by the average geometric mean (Attune NxT–YL2-A, Ex. 560 nm, Em. 610 nm) of Nile red stained, empty vector (EV104) transformed *E. coli* was removed, and these data were normalized to Native *phaCAB*-engineered *E. coli. In vivo* PHAs production from whey permeate in *phaCAB*-engineered *E. coli.* PHAs were purified from these PHAs production cultures and measured as (**c**) PHAs content [weight (wt) % of cell dry weight (CDW)] and (**d**) PHAs production (g/l). Error bars indicate the standard error of the mean. Student’s *t*-test, ***P < 0.001, and ****P < 0.0001, *n* = 3–4.

Qualitative analysis of Nile red stained *E*. *coli* confirmed PHAs polymer production in Native, C104 and C101 *phaCAB*-engineered *E. coli* (Figure 4a). Whereas, negative control (EV104 and EV101) and **Δ**PhaC_C319A *phaCAB*-engineered *E. coli* did not generate PHAs granules (Figure 4a). In addition to the plate assays, a semi-quantitative Nile red flow cytometry analysis of *in vivo* PHAs production was also carried out (Figure 4b, Supplementary Figure S6–S8 and Table S5). For these analyses, a gating strategy was implemented that excluded cell doublets and distinguished between negatively and positively Nile red stained cell populations (Supplementary Figure S6 and Table S4). Post gating, the background signal of negative control cells (EV104 or EV101, as appropriate) was removed, and these data were normalized to the Nile red fluorescence of Native *phaCAB*-engineered *E. coli.* Nile red fluorescence levels were on average 2.18 ± 0.05 fold-higher in C104 *phaCAB*-engineered and 1.727 ± 0.09 fold-higher in C101 *phaCAB*-engineered *E. coli* than Native *phaCAB*-engineered *E. coli* levels (Figure 4b, Supplementary Figure S7 and S8) indicating that PHAs content was highest in C104 *phaCAB*-engineered *E. coli*. Therefore, we focused our *in vivo* PHAs production experiments towards using the least (Native) and most active (C104) *phaCAB* operons that we had tested.

*In vivo* PHAs production experiments involving *phaCAB*-engineered *E. coli* were carried out in whey permeate production media and post-production, a sodium hypochlorite-based method was used to purify PHAs content from these strains. PHAs production from whey permeate was almost three times higher in C104 *phaCAB*-engineered (5.10 ± 0.35 g/l, ∼48.9 mM) cultures than Native PHAs production cultures (1.83 ± 0.07 g/l, ∼17.6 mM) (Figure 4d and Supplementary Table S6). PHAs production was also validated using GC-MS (Supplementary Table S7). Similarly, PHAs content expressed as % weight of cell dry mass was also significantly higher in C104 *phaCAB*-engineered cultures (68.96 ± 4.91%) than Native PHAs production cultures (16.14 ± 0.68%) (Figure 4c and Supplementary Table S6). Although the reaction scales, whey concentrations and PHAs production yields differ between these *in vivo* and *in vitro* cell-free assays, it is clear that the C104 *phaCAB* operon, and its derivative C104 **Δ**PhaC_C319A were more active than the Native operon as observed in both cell-free and *in vivo* assays. This therefore illustrates how cell-free prototyping approaches can complement *in vivo* workflows for prototyping PHAs biosynthetic operons.

## 4 Summary and conclusions

Despite the environmental consequences that are associated with the mass production of oil-derived plastics, global demand is likely to continue to increase unless viable economic alternatives are developed (1, 3). The PHAs are a family of biodegradable biopolymers, that could potentially be used as sustainable alternatives to replace several widely used oil-derived plastics (e.g. polypropylene). However, PHAs are currently more expensive to produce than oil-derived plastics, which has hampered their adoption. Therefore, more efficient PHAs production processes would be desirable. An array of advancements in synthetic biology and metabolic engineering have already led to improvements in PHAs production though we anticipate that cell-free metabolic engineering approaches, several of which have been underutilized in PHAs research (e.g. cell-free TX-TL prototyping), could lead to additional innovations in PHAs production strategies. Indeed, cell-free metabolic engineering approaches have already been used to prototype biosynthetic pathways in other contexts (49). Yet, interestingly, cell-free TX-TL systems have not previously been used to prototype PHAs biosynthetic operons. This may in part relate to the challenges associated with detecting PHAs and PHAs monomers produced in small scale cell-free reactions. In order to overcome this and to accelerate the adoption of cell-free TX-TL prototyping platforms in PHAs research, several cell-free TX-TL approaches for characterizing PHAs biosynthetic pathways were developed. *E. coli* MG1655 cell-free prototyping reactions were customized with whey permeate as an energy source. Whey permeate is an industrial waste that has been previously used as a low-cost feedstock for optimizing *in vivo* PHAs production. The inclusion of an optimal concentration of whey permeate enhanced relative *in vitro* protein production and subsequent experiments also demonstrated, for the first time, the production and GC-MS detection of 3HB (a PHAs monomer) within cell-free TX-TL reactions. pH and Acetyl-CoA assays were also used to demonstrate that additional insights into the activities of PHAs biosynthetic pathways can also be gained through carrying out cell-free TX-TL prototyping assays. Therefore, these data demonstrate that suitably customized cell-free TX-TL systems can be used to characterize PHAs biosynthetic operons within a metabolic context that relates to *in vivo* production. Additionally, we also demonstrated that coupled cell-free biotransformation with TX-TL strategies can be used to screen for useful metabolite recycling enzymes for enhancing *in vivo* PHAs production. As an exemplar, we *in vitro* expressed and characterized an Acetyl-CoA recycling enzyme (*pct*) within *phaCAB-*engineered biotransformation cell extracts.

**Figure 5.**
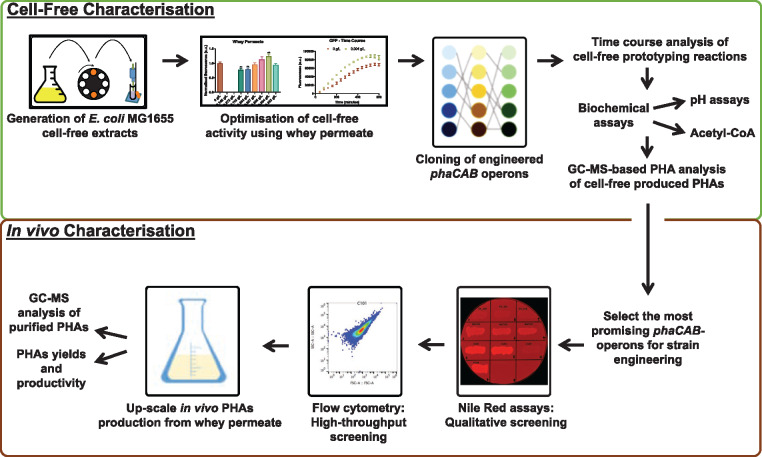
Cell-free and *in vivo* characterization of PHAs production. Cell-free prototyping approaches complement *in vivo* strategies for characterizing Polyhydroxyalkanoates (PHAs) production from whey permeate.

More broadly, we envision that these types of cell-free metabolic engineering approaches could conceivably be used in combination with *in vivo* strategies for optimizing PHAs production (Figure 5). Ultimately, the continuing development of cell-free metabolic engineering approaches may lead to desirable innovations in PHAs production that enhance their potential as sustainable alternatives to oil-derived plastics.

## Supplementary Material

Supplementary Data
