# Supplementary material for: Cell-free prototyping strategies for enhancing the sustainable production of polyhydroxyalkanoates bioplastics
Source: Synth Biol (Oxf). 2018 Sep 4;3(1):ysy016. doi: 10.1093/synbio/ysy016 (PMC7445755; doi:10.1093/synbio/ysy016)
Supplement: Supplementary Data [file ysy016_supp.docx]

**Supplementary:**

**Cell-free prototyping strategies for enhancing the sustainable production of polyhydroxyalkanoates bioplastics**

Richard Kelwick^1,2#^, Luca Ricci^1,2,3#^, Soo Mei Chee^4^, David Bell^4^, Alexander J. Webb^1,2^ and Paul S. Freemont^1,2,4*^

^1^Centre for Synthetic Biology and Innovation, Imperial College London, London, SW7 2AZ, UK. ^2^Section of Structural Biology, Department of Medicine, Imperial College London, London, SW7 2AZ, UK. ^3^Department of Life Sciences and Systems Biology, University of Turin. ^4^SynbiCITE, Department of Medicine, Imperial College London, London, SW7 2AZ, UK.

^#^Joint First Authors

*To whom correspondence should be addressed:

Paul Freemont

Section of Structural Biology

Department of Medicine

Sir Alexander Fleming Building

South Kensington Campus

Exhibition Road

London

SW7 2AZ

UK

Email: p.freemont@imperial.ac.uk

Tel: +44 (0) 207 594 5327

**Supplementary Figure 1. SNARF-5F pH calibration curve. (A)** The fluorescence ratio (excitation 514 nm and the ratio of two different emissions, 580 nm and 640 nm) of SNARF-5F (100 μM final concentration) pH sensitive dye was measured using a range of different 100 mM Tris buffers that were set, using 0.5 M acetic acid, at a range of defined pH strengths. **(B)** Third order polynomial fitting for extrapolating pH from the fluorescence emission ratio of SNARF-5F in the different pH calibration buffer solutions.

**Supplementary Figure 2. Whey permeate enhances cell-free protein synthesis.**

Endpoint (5 h) analysis of cell-free GFP production in 30 µl cell-free transcription-translation reactions with 0 or 0.004 g/L whey permeate. The background fluorescence of cell-free reactions using negative control plasmid (EV101) were subtracted and these data were normalised to the relative fluorescence of cell-free reactions containing 0 g/L whey permeate. Error bars denote standard error of the mean. Student t-test, *P<0.05.

**Supplementary Figure 3. Lactose enhances cell-free protein synthesis. (a)** Endpoint (5 h) analysis of cell-free GFP production in cell-free transcription-translation reactions with 0-4.836 g/L lactose. The background fluorescence of cell-free reactions using negative control plasmid (EV101) were subtracted and these data were normalised to the relative fluorescence of cell-free reactions containing 0 g/L lactose. **(b)** Time-course analysis of pH in cell-free reactions which included 0-4.836 g/L of lactose. Control reactions were lactose without cell extract. Error bars denote standard error of the mean. Student t-test, **P<0.01.

**Supplementary Figure 4. GC-MS 3HB calibration curve.** A commercial 3-hydroxybutyrate (3HB) standard (Sigma Aldrich, USA) was dissolved in methanol (Sigma Aldrich, USA) at several different concentrations between the range of 0 µM – 200µM in serial dilutions to generate a 3HB calibration curve. These 3HB calibration curves were generated in each GC-MS run of cell-free samples and a representative calibration curve is shown here.

**Supplementary Figure 5. Acetyl-CoA calibration curve.** The Acetyl-CoA calibration curve was setup, according to the manufacturer’s instructions, using reagents from the PicoProbe Acetyl CoA Assay Kit (Abcam, Cambridge, UK, Catalogue No. #87546). The fluorescence readings were measured using a Clariostar (BMG, UK) plate reader (Ex535-10/Em. 587-10).

**
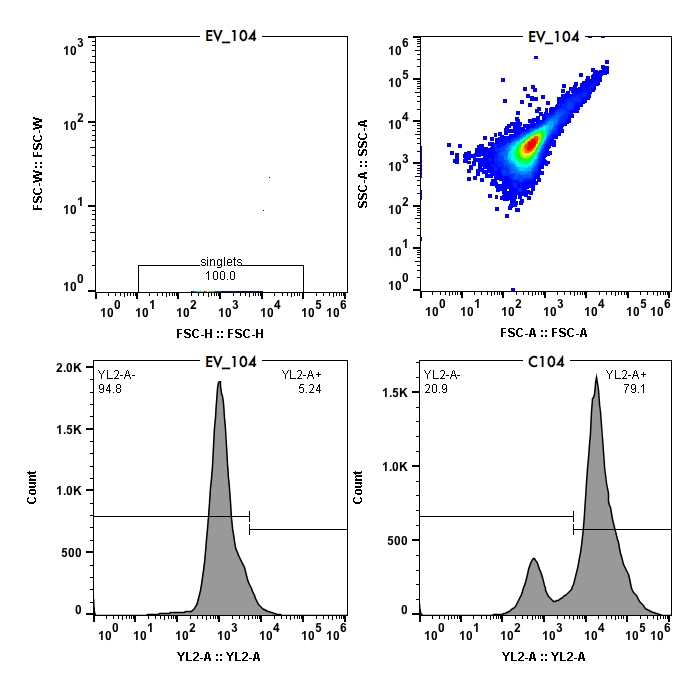
**

**Supplementary Figure 6. Flow gating strategy for doublet discrimination and Nile Red stained positive cell populations. (Top Left)** Representative gating strategy used to remove *E. coli* doublets (or cell clumps) from all flow cytometry analysis. **(Top right).** Representative forward scatter (FSC) and side scatter (SSC) contour plot of a singlet *E. coli* population (EV104 strain). Representative fluorescence histograms of negative control EV104 strain **(Bottom left)** and C104 *phaCAB-*engineered *E. coli* **(Bottom right)** analyses post-doublet discrimination. PHA content was determined via flow cytometry (Attune NxT) and FlowJo software (v 10.1) analysis of Nile Red staining (YL2-A, Ex. 560 nm, Em. 610 nm). The gating strategies used to distinguish between positive (YL2-A+) and negative (YL2-A-) Nile Red stained populations are also shown in the bottom two panels.


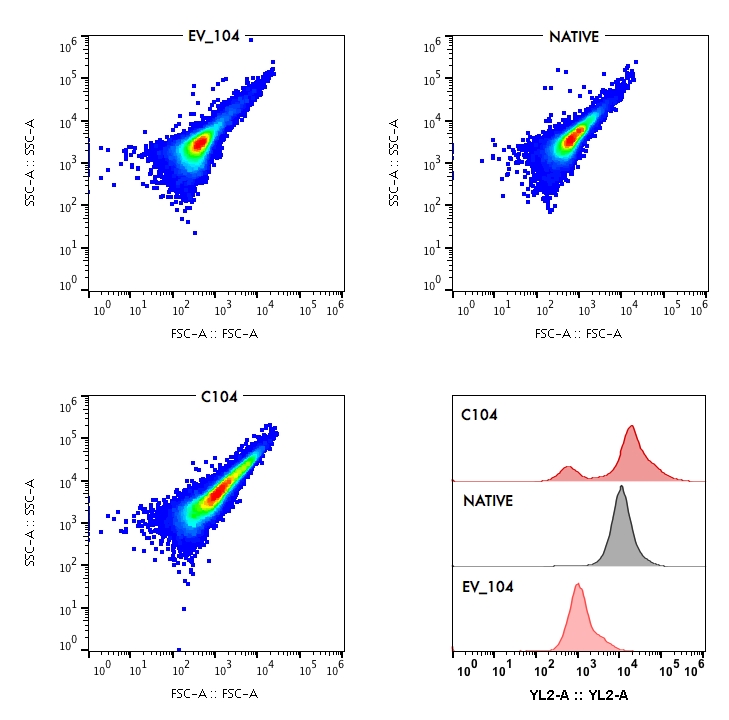


**Supplementary Figure 7. Flow cytometry analysis of PHAs production from whey permeate in *phaCAB* engineered *E. coli*.** *E. coli* MG1655 transformed with either an empty vector (EV104) or a *phaCAB* operon (Native or C104) were grown overnight at 37^o^C in 5 ml of 2xYT+P media supplemented with 34 μg/ml Chloramphenicol. The resultant cultures were diluted to an OD_600nm_ of 0.8 in 6 ml of PHA production media (2xYT+P supplemented with 120.48 g/L whey permeate and 34 μg/ml Chloramphenicol) and were cultured at 37^o^C for 24 hours. In order to assess PHA content, cultures were Nile Red stained and analysed via flow cytometry (Attune NxT) and FlowJo (v 10.1) analysis software. These figures are representative forward scatter (FSC) / side scatter (SSC) contour plots and fluorescence histograms (YL2-A, Ex. 560 nm, Em. 610 nm) of the data shown in Figure 4b.

**Supplementary Figure 8. Flow cytometry analysis of PHAs production in *phaCAB* engineered *E. coli*.** *E. coli* MG1655 transformed with either an empty vector (EV101) or a *phaCAB* operon (Native or C101) were grown overnight at 37^o^C in 5 ml of 2xYT+P media supplemented with 34 μg/ml Chloramphenicol. The resultant cultures were diluted to an OD_600nm_ of 0.8 in 6 ml of PHA production media (2xYT+P, 3% glucose (w/v) supplemented with 34 μg/ml Chloramphenicol) and were cultured at 37^o^C for 24 hours. In order to assess PHAs content, cultures were Nile Red stained and analysed via flow cytometry (Attune NxT) and FlowJo (v 10.1) analysis software. These figures are representative forward scatter (FSC) / side scatter (SSC) contour plots and fluorescence histograms (YL2-A, Ex. 560 nm, Em. 610 nm). The background signal, as determined by the average geometric mean (YL2-A) of Nile Red stained, empty vector (EV101) transformed *E. coli* was removed and these data were normalised to native *phaCAB*-engineered *E. coli.* Error bars indicate the standard error of the mean of three independent replicates. Student t-test, ^*^P<0.05.

**Supplementary Table 1. Bacterial strains and plasmids used in this study.**

| **Strain** | **Relevant features** | **Reference** |
| --- | --- | --- |
| MG1655 | K-12 F^–^ λ^–^ *ilvG*^–^ *rfb-50* *rph-1* | 1 |
| NEB10-beta | Δ(*ara -leu*) 7697 *araD139 fhuA* Δ*lacX74 galK16 galE15 e14-*ϕ*80*d*lacZ*Δ*M15* *recA1 relA1endA1 nupG rpsL* (StrR) *rph spoT1* Δ(*mrr-hsdRMS-mcrBC*); Cloning strain | New England Biolabs |
| TOP10 | F- *mcrA* Δ(*mrr-hsd*RMS-*mcr*BC) ϕ 80*lacZ*ΔM15 Δ*lac*X74 *rec*A1 *ara*D139 Δ(*ara-leu*)7697 *gal*U *gal*K *rps*L(StrR) *end*A1 *nup*G; Cloning strain | Life Technologies |
| pRK1 | TOP10 pSB1C3-BBa_I13504; RBS B0034, GFPmut3b and terminators B0010 and B0012, [BBa_I13504]; CamR.  **TOP10 *E. coli* strain transformed with BBa_I13504 in pSB1C3 plasmid backbone.** | 2 |
| pRK21 | TOP10 pSB1C3-J23101; J23101 promoter (BBa_J23101), [EV101]; CamR.  **TOP10 *E. coli* strain transformed with J23101 constitutive promoter in pSB1C3 plasmid backbone.** | This study |
| pRK3 | MG1655 pSB1C3-J23101; J23101 promoter (BBa_J23101), [EV101]; CamR.  **MG1655 *E. coli* strain transformed with J23101 constitutive promoter in pSB1C3 plasmid backbone.** | This study |
| pLR036 | NEB10-beta pSB1C3-J23101-I13504; J23101 promoter (BBa_J23101), RBS B0034, GFPmut3b and terminators B0010 and B0012, [101_GFP]; CamR.  **NEB10-beta *E. coli* strain transformed with *gfpmut3b* expression plasmid (J23101 constitutive promoter, RBS B0034, *gfpmut3b* and terminators B0010 and B0012) on pSB1C3 plasmid backbone.** | This study |
| pRK4 | MG1655 pSB1C3-J23104-B0034; J23104 promoter and RBS B0034 (BBa_K608002), [EV104]; CamR.  **MG1655 *E. coli* strain transformed with J23104 constitutive promoter and RBS B0034 in pSB1C3 plasmid backbone.** | 3 |
| pRK51 | MG1655 pSB1C3-*phaCAB*; native *phaCAB* operon (BBa_K934001), [Native]; CamR.  **MG1655 *E. coli* strain transformed with Native *phaCAB* operon (BBa_K934001) on pSB1C3 plasmid backbone.** | 3 |
| pRK61 | MG1655 pSB1C3-J23104-B0034-*phaCAB*; *phaCAB* under the control of the constitutive J23104 promoter (BBa_K1149052), [C104]; CamR.  **MG1655 *E. coli* strain transformed with C104 *phaCAB* operon (BBa_K1149052) on pSB1C3 plasmid backbone.** | 3 |
| pRK7 | NEB10-beta pSB1C3-J23104-B0034-*phaCAB*; *phaCAB* under the control of the constitutive J23104 promoter (BBa_K1149052), inactive PhaC (cysteine-319-alanine) [C104 △PhaC_C319A]; CamR.  **NEB10-beta *E. coli* strain transformed with C104 *phaCAB* operon (BBa_K1149052) with an inactive PhaC on pSB1C3 plasmid backbone.** | This study |
| pRK8 | MG1655 pSB1C3-J23104-B0034-*phaCAB*; *phaCAB* under the control of the constitutive J23104 promoter (BBa_K1149052), inactive PhaC (cysteine-319-alanine) [C104 △PhaC_C319A]; CamR.  **MG1655 *E. coli* strain transformed with C104 *phaCAB* operon (BBa_K1149052) with an inactive PhaC on pSB1C3 plasmid backbone.** | This study |
| pRK9 | TOP10 pSB1A2-pT7; pT7 promoter (BBa_I719005), [pT7 only]; AmpR.  **TOP10 *E. coli* strain transformed with pT7 promoter (BBa_I719005) on pSB1A2 plasmid backbone.** | This study |
| pRK10 | TOP10 pSB1A2-pT7-I13504; pT7 promoter (BBa_I719005), RBS B0034, GFPmut3b and terminators B0010 and B0012 [pT7_GFPmut3b]; AmpR.  **TOP10 *E. coli* strain transformed with inducible *gfpmut3b* expression plasmid (pT7 promoter BBa_I719005, RBS B0034, *gfpmut3b* and terminators B0010 and B0012) on pSB1A2 plasmid backbone.** | This study |
| pRK11 | NEB10-beta pCR-Blunt-PCT; propionyl CoA transferase (PCT) gene from Clostridium propionicum [PCT-Blunt]; KanR.  **NEB10-beta *E. coli* transformed with PCT gene that was cloned into pCR-Blunt vector.** | This study |
| pRK12 | NEB10-beta pSB1A2-pT7-PCT; pT7 promoter (BBa_I719005), RBS B0034, PCT and terminators B0010 and B0012 [pT7_PCT]; AmpR.  **NEB10-beta *E. coli* strain transformed with an inducible *pct* expression plasmid (pT7 promoter BBa_I719005, RBS B0034, *pct* and terminators B0010 and B0012) on pSB1A2 plasmid backbone.** | This study |
| pRK13 | NEB 10-beta pSB1C3-*phaCAB*; native *phaCAB* operon (BBa_K934001), inactive PhaC (cysteine-319-alanine) [Native △PhaC_C319A]; CamR.  **NEB10-beta *E. coli* strain transformed with Native *phaCAB* operon (BBa_K934001) with an inactive PhaC on pSB1C3 plasmid backbone.** | This study |
| pRK14 | MG1655 pSB1C3-J23101-B0034-*phaCAB*; *phaCAB* under the control of the constitutive J23101 promoter, [C101]; CamR.  **MG1655 *E. coli* strain transformed with C101 *phaCAB* operon on pSB1C3 plasmid backbone. C101 is the same as C104 except that the operon is driven by a J23101 constitutive promoter.** | This study |
| pRK15 | NEB 10-beta pSB1C3-J23101-B0034-*phaCAB*; *phaCAB* under the control of the constitutive J23101 promoter, inactive PhaC (cysteine-319-alanine) [C101 △PhaC_C319A]; CamR.  **NEB10-beta *E. coli* strain transformed with C101 *phaCAB* operon with an inactive PhaC on pSB1C3 plasmid backbone.** | This study |
| S_RK001 | *E.coli* MG1655 pRK4 [EV104] | 3 |
| S_RK002 | *E.coli* MG1655 pRK51 [Native] | 3 |
| S_RK003 | *E.coli* MG1655 pRK61 [C104] | 3 |
| S_RK004 | *E.coli* MG1655 pRK8 [C104 △PhaC_C319A] | This study |
| S_RK005 | *E.coli* NEB10-beta pRK13 [Native △PhaC_C319A] | This study |
| S_RK006 | *E.coli* MG1655 pRK14 [C101] | This study |
| S_RK007 | *E.coli* NEB10-beta pRK15 [C101 △PhaC_C319A] | This study |
| S_RK008 | *E.coli* MG1655 pRK3 [EV101] |  |

Note: “BBa_, B00XX, J2310X and pSB1C3” refer to iGEM parts registry BioBricks and relevant sequences see: <http://parts.igem.org/Main_Page>.

Plasmid maps and genbank sequences provided as supplementary.

**Supplementary Table 2. Oligonucleotide primers used in this study.**

| **Number** | **Name** | **Sequence** |
| --- | --- | --- |
| **Primers for cloning** | | |
| RK007 | PhaCCys319Ala_F | ggctttgcagtcggtggcactatcg |
| RK008 | PhaCCys319Ala_R | accgactgcaaagccgagtacgttaatc |
| RK009 | T7_INF_FWD | TACTAGAGCCAGGCATCAAATAAAACG |
| RK010 | T7_INF_REV | CTAGTATTTCTCCTCTTTCTCTAG |
| RK011 | F1_PCT-FW | GAGGAGAAATACTAGATGCGTAAAGTTCCGATTATT |
| RK012 | F1_PCT-RV | TGCCTGGCTCTAGTATTAGCTTTTCATTTCTTTCAGG |
| **Primers for sequencing** | | |
| RK013 | VF2 | TGCCACCTGACGTCTAAGAA |
| RK014 | VR | ATTACCGCCTTTGAGTGAGC |
| RK015 | PhaCseq1 | TGGCAGGCGATGGCGGAAG |
| RK016 | PhaB_F | ATGACTCAGCGCATTGCGTATGTGA |
| RK017 | PhaCSeq2 | CTATGGCAACGCGCGCTACC |
| RK018 | phaA2_Rv | TGGCATAGCTCTTGATCGTG |
| RK019 | PhaASeq1 | GTTCCCTCCCGTTTCC |
| RK020 | PhaBSeq1 | GCGACGATAACGAAGCC |
| RK021 | M13 FWD | GTAAAACGACGGCCAG |
| RK022 | M13 Reverse | CAGGAAACAGCTATGAC |
| RK023 | PhaC_seq4_F | TCTGCTGCGCGGTCTGG |
| RK024 | CH101F | CTAGGTATTATGCTAGCTACTAGAGAAAGAGG |
| RK025 | CH101R | GACTGAGCTAGCTGTAAACTCTAGAAGCGGCC |

**FASTA files included as supplementary.**

**Supplementary Table 3. Molkolac instant demineralised whey permeate composition.**

| **Component** | **Values per 100g** |
| --- | --- |
| Fat | 0.08 g |
| Carbohydrates | 84.13 g |
| Protein | 2.57 g |
| Calcium | 488 mg |
| Magnesium | 108 mg |
| Potassium | 1924 mg |
| Sodium | 546 mg |
| Chloride | 921 mg |
| Phosphorus | 590 mg |

These whey composition data were provided by the supplier (Orchard Valley Food Ingredients, UK). 120.48 g/L whey permeate is equivalent to ~100 g/L carbohydrates.

**Supplementary Table 4. GC-MS analysis of 3HB production in coupled cell-free biotransformation with cell-free transcription and translation (*pct* gene) reactions.**

| **Cell-free reaction** | **3HB (µM)** |
| --- | --- |
| -T7 | 26.18 ±4.188 |
| +T7 | 32.84 ±4.327 |

n=3, student t-test p=0.34 (not significant).

**Supplementary Table 5. Flow cytometry analysis of *in vivo* PHAs production from whey permeate.**

| **Sample** | **Total Number of Cells** | **Geomean (YL2-A)** | **YL2-A+ cells (%)** | **Geomean (YL2-A+)** | **YL2-A- cells (%)** | **Geomean (YL2-A -)** |
| --- | --- | --- | --- | --- | --- | --- |
| **EV104** | 60739 | 757 | 2.73 | 8654 | 97.3 | 707 |
| **EV104** | 53786 | 1126 | 5.24 | 7905 | 94.8 | 1011 |
| **EV104** | 60473 | 2245 | 15.3 | 8254 | 84.7 | 1774 |
| **Native** | 48075 | 8038 | 81.5 | 10595 | 18.5 | 2385 |
| **Native** | 60185 | 9310 | 87.1 | 11485 | 12.9 | 2255 |
| **Native** | 60247 | 10438 | 90.2 | 12500 | 9.77 | 1974 |
| **C104** | 63045 | 11514 | 78.6 | 24398 | 21.4 | 735 |
| **C104** | 61845 | 11163 | 79.1 | 23189 | 20.9 | 697 |
| **C104** | 50313 | 9644 | 76.1 | 22831 | 23.9 | 619 |

**Supplementary Table 6. Polyhydroxyalcanoates (PHAs) production**.

| **Carbon source** | **E. coli Strain** | **PHA produced (g/L)** | **PHA content (wt. % of cell dry weight)** |
| --- | --- | --- | --- |
| **Glucose** | Native | 0.73 ± 0.03 | 18.64 ± 0.73 |
|  | C104 | 2.00 ± 0.21 ** | 45.35 ± 4.13 ** |
| **Whey permeate** | Native | 1.83 ± 0.07 | 16.14 ± 0.68 |
|  | C104 | 5.10 ± 0.35 *** | 68.96 ± 4.91 *** |

Statistical analysis in comparison to native *phaCAB* engineered *E. coli*. Student t-test, ^*^P<0.05, ^**^P<0.01, ***P<0.001, and ****P<0.0001.

**Supplementary Table 7. Gas chromatography–mass spectrometry (GC-MS) analysis of purified PHA samples.**

| **Strain** | **Feedstock** | **Polymer produced** | **Me-3HB peak area** |
| --- | --- | --- | --- |
| Native | Whey Permeate | P(3HB) | 771576 |
| Native | Whey Permeate | P(3HB) | 506052 |
| Native | Whey Permeate | P(3HB) | 696400 |
| C104 | Whey Permeate | P(3HB) | 914566 |
| C104 | Whey Permeate | P(3HB) | 1095772 |
| C104 | Whey Permeate | P(3HB) | 427680 |

**Supplementary References**

1. Blattner, F. R. *et al.* The complete genome sequence of Escherichia coli K-12. *Science* **277,** 1453–62 (1997).

2. Kelwick, R., Webb, A. J., MacDonald, J. T. & Freemont, P. S. Development of a Bacillus subtilis cell-free transcription-translation system for prototyping regulatory elements. *Metab. Eng.* (2016). doi:10.1016/j.ymben.2016.09.008

3. Kelwick, R. *et al.* A forward-design approach to increase the production of poly-3-hydroxybutyrate in genetically engineered Escherichia coli. *PLoS One* **10,** e0117202 (2015).
